# PSA Density Help to Identify Patients With Elevated PSA Due to Prostate Cancer Rather Than Intraprostatic Inflammation: A Prospective Single Center Study

**DOI:** 10.3389/fonc.2021.693684

**Published:** 2021-05-20

**Authors:** Salvatore M. Bruno, Ugo G. Falagario, Nicola d’Altilia, Marco Recchia, Vito Mancini, Oscar Selvaggio, Francesca Sanguedolce, Francesco Del Giudice, Martina Maggi, Matteo Ferro, Angelo Porreca, Alessandro Sciarra, Ettore De Berardinis, Carlo Bettocchi, Gian Maria Busetto, Luigi Cormio, Giuseppe Carrieri

**Affiliations:** ^1^ Department of Urology and Renal Transplantation, University of Foggia, Foggia, Italy; ^2^ Department of Pathology, University of Foggia, Foggia, Italy; ^3^ Department of Urology, Sapienza Rome University, Rome, Italy; ^4^ Department of Urology, European Institute of Oncology (IEO) IRCCS, Milan, Italy; ^5^ Department of Urology, Veneto Institute of Oncology (IOV) IRCCS, Padua, Italy

**Keywords:** PSA density, PSA, prostate cancer, Irani score, prostate inflammation

## Abstract

The association between PSA density, prostate cancer (PCa) and BPH is well established. The aim of the present study was to establish whether PSA density can be used as a reliable parameter to predict csPCa and to determine its optimal cutoff to exclude increased PSA levels due to intraprostatic inflammation. This is a large prospective single-center, observational study evaluating the role of PSA density in the discrimination between intraprostatic inflammation and clinically significant PCa (csPCa). Patients with PSA ≥ 4 ng/ml and/or positive digito-rectal examination (DRE) and scheduled for prostate biopsy were enrolled. Prostatic inflammation (PI) was assessed and graded using the Irani Scores. Multivariable binary logistic regression analysis was used to assess if PSA density was associated with clinically significant PCa (csPCa) rather than prostatic inflammation. A total of 1988 patients met the inclusion criteria. Any PCa and csPCa rates were 47% and 24% respectively. In the group without csPCa, patients with prostatic inflammation had a higher PSA (6.0 vs 5.0 ng/ml; p=0.0003), higher prostate volume (58 vs 52 cc; p<0.0001), were more likely to have a previous negative biopsy (29% vs 21%; p=0.0005) and a negative DRE (70% vs 65%; p=0.023) but no difference in PSA density (0.1 vs 0.11; p=0.2). Conversely in the group with csPCa, patients with prostatic inflammation had a higher prostate volume (43 vs 40 cc; p=0.007) but no difference in the other clinical parameters. At multivariable analysis adjusting for age, biopsy history, DRE and prostate volume, PSA density emerged as a strong predictor of csPCA but was not associated with prostatic inflammation. The optimal cutoffs of PSA density to diagnose csPCa and rule out the presence of prostatic inflammation in patients with an elevated PSA (>4 ng/ml) were 0.10 ng/ml^2^ in biopsy naïve patients and 0.15 ng/ml^2^ in patients with a previous negative biopsy. PSA density rather than PSA, should be used to evaluate patients at risk of prostate cancer who may need additional testing or prostate biopsy. This readily available parameter can potentially identify men who do not have PCa but have an elevated PSA secondary to benign conditions.

## Introduction

“There is moderate certainty that the benefits of prostate-specific antigen (PSA)-based screening for prostate cancer (PCa) do not outweigh the harms”. In 2012, based on the results of two large-scale randomized clinical trials (RCT’s), the United States Preventive Services Task Force (USPSTF) issued a grade D recommendation discouraging PSA-based screening (1). Since this strategy could lead to a substantial number of men with aggressive disease being missed, the USPSTF issued an updated statement in 2017. While the grade of recommendation remained unchanged for men over 70 years old, it has been changed from D to C in men aged 55-69 years old. PSA testing should be offered to selected man depending on individual circumstances and counseling patients about the potential benefits and harms of PSA-based screening, as this might be associated with a small survival benefit (2). Similarly, European association of urology (EAU) Guidelines suggest offering an individualized risk-adapted strategy for early detection to a well-informed man and a life-expectancy of at least 10 to 15 years (3).

The major limitations of screening using PSA have been underlined in a Cochrane review of five available RCT’s. Screening is associated with an increased diagnosis of PCa, with detection of more localized disease and less advanced PCa with no benefit on PCa-specific and overall survival (4).

Still, screening for PCa is one of the most controversial topics in the urological literature. PSA is not specific for PCa. Several other benign conditions can cause a man’s PSA level to rise such as inflammation and benign prostatic hyperplasia (BPH). To date there is no evidence that inflammation or BPH leads to prostate cancer, but it is possible for a man to have one or both conditions and to develop PCa as well.

In this scenario PSA density, expressed as the PSA value (in ng/ml) divided by prostate volume (in CC), can potentially identify men who do not have PCa but have an elevated PSA secondary to benign conditions.

The association between PSA density, PCa and BPH is well established (5, 6). The aim of the present study was to establish whether PSA density can be used as a reliable parameter to predict csPCa and to determine its optimal cutoff to exclude increased PSA levels due to intraprostatic inflammation.

## Materials and Methods

### Study Population

This is a prospective single center, observational study evaluating the role of intraprostatic inflammation in prostate cancer screening and treatment. From March 2014 to December 2019, all patients referred to our institution to perform prostate biopsy (PBx) for a PSA ≥ 4 ng/ml and/or positive digital rectal examination (DRE) were enrolled, and data were prospectively entered into our database. Sample size was not computed *a priori* and according to the protocol we enrolled all eligible patients during the study period. Patients on active surveillance with a previous positive biopsy (n=87), men receiving 5 alfa-reductase inhibitors (5-ARIs) (n=65), or who had previously undergone invasive treatment for BPH (n=36), or with dwelling urethral catheters (n=22) and man with PSA > 20 ng/ml (n=96) were excluded. The study protocol was approved by the University of Foggia Ethics Committee and written informed consent to take part was given by all participants (Decision n. 152/CE/2014 of September 03, 2014; Ethical Committee at the University Hospital “Ospedali Riuniti”, Foggia, Italy).

All patients underwent PSA measurement before DRE and transrectal ultrasound (TRUS). Uroflowmetry (UFM) was carried out with “Flowline II” before PBx, waiting for the patient to report a strong sensation to void. Peak flow rate (Qmax) and ultrasound post void residual volume (PVR) were annotated. Additionally, all patients filled the International Prostate Symptom Score (IPSS) survey (7). Following local non-infiltrative anesthesia (8), prostate biopsy was performed according to our 18 cores standard biopsy template (9) under TRUS guidance (BK Medical Flex Focus 500) and using an 18 gauge/25 cm biopsy needle (Bard Max-Core). As per our protocol, patients had a single shot of cefazolin right before the procedure or a course of quinolones or cotrimoxazole starting the night before the procedure.

### Pathological Examination

A senior uropathologist (FS) prospectively evaluated all PBx specimens according to the International Society of Urological Pathology (ISUP) recommendations (10). Additionally, prostatic inflammation (PI) was assessed and graded using the Irani Scores (5) subsequently validated by Sciarra et al. (11). Specifically, the inflammatory infiltration was graded as “G0” = no inflammatory cells, “G1” = scattered inflammatory cell infiltrate within the stroma without lymphoid nodules, “G2” = nonconfluent lymphoid nodules and “G3” = large inflammatory areas with confluence of infiltrate. Inflammatory aggressiveness was graded as “A0” = no contact between inflammatory cells and glandular epithelium (epithelium cells lining acini and ducts), “A1” = contact between inflammatory cell infiltrate and glandular epithelium, “A2” = interstitial inflammatory infiltrate associated with a clear but limited (less than 25% of the examined material) glandular epithelium disruption and “A3” = glandular epithelium disruption on more than 25% of the examined material. Irani total score was computed as the sum of the Irani G and Irani A scores. Grading did not include the types of inflammatory cells (polymorphonuclear leukocytes, lymphocytes, monocytes or plasma cells).

### Statistical Analysis

Outcomes of this study were clinically significant PCa (csPCa) defined as Gleason Grade Group (GGG) ≥ 2(≥3+4) and presence of prostatic inflammation defined as Irani total score ≥2. Variables of interest were available in all patients included in the study.

Descriptive statistics was performed for the overall population and according to biopsy results. Continuous variables were reported as median and interquartile range and compared by the Mann-Whitney U-test, whereas categorical variables were reported as rates and tested by the Fisher’s exact test or the chi-square test, as appropriate.

Since inflammation and csPCa often coexists, we stratified patients in four groups (both present, both absent, prostatic inflammation without csPCa and csPCa without inflammation) and we compared clinical characteristics in patients with and without inflammation but no csPCa, and patients with and without inflammation but diagnosed with csPCa. Multivariable binary logistic regression analysis was then used to assess if PSA density was associated with csPCa rather than prostatic inflammation. Age, biopsy history, DRE and PSA density were included in the multivariable model. In order to provide clinicians with a readily available tool to evaluate risk of elevated PSA due to csPCa, rather than inflammation, we graphically presented the histological findings of patients with a PSA >4 ng/ml according to PSA density groups and biopsy history. Finally, the actual probability of biopsy-detected prostate cancer and prostatic inflammation for a given PSA density value were calculated using locally weighted scatterplot (“lowess”) smoothing.

Statistical analyses were performed using Stata-SE 15 (StataCorp LP, College Station, TX, USA) using the following syntax: kwallis, chi2, logistic, graph bar. All tests were 2-sided with a significance level set at p<0.05.

## Results

### Descriptive Characteristics of the Overall Population

A total of 1988 patients met the inclusion criteria. Clinical characteristics and histopathological results of the overall population and according to biopsy results are shown in **Table 1**. The majority of patients (78% n=1547) were biopsy naïve. Any PCa and csPCa rates were 47% and 24% respectively. High grade inflammation (Irani G 2-3) was present in 639 (32.1%) patients and 984 (49.5%) patients had highly aggressive inflammation (Irani A 1-2-3). Patients diagnosed with any PCa (GGG1) and csPCa (GGG≥2) were older, had greater PSA and PSA density suspicious DRE and Qmax, but lower prostate volume, PVR and IPSS than those without cancer. Interestingly, high- grade inflammation (Irani G 2-3) was significantly more common in patients with benign prostate than in those with any PCa and csPCa, and the same applied to highly aggressive inflammation (Irani A 1-2-3).

**Table 1:** Clinical characteristics and histopathological results of the overall population and according to biopsy results.

Variable	Overall population N=1988	Negative Biopsy N=1045 (52.6%)	GGG 1 N=458 (23.0%)	GGG ≥2 N=485 (24.4%)	P Value
**Age**	67 (61, 72)	65 (60, 70)	67 (62, 72)	70 (65, 75)	<0.0001
**PSA**	6.0 (4.6, 8.7)	5.9 (4.6, 8.1)	5.6 (4.4, 8.5)	6.7 (5.0, 10.5)	<0.0001
**Biopsy History, n (%)**					
Biopsy Naive	1547 (77.8%)	745 (71.3%)	371 (81.0%)	431 (88.9%)	<0.0001
Prev. Negative	441 (22.2%)	300 (28.7%)	87 (19.0%)	54 (11.1%)	
**DRE, n (%)**					
Negative	1232 (62.0%)	724 (69.3%)	298 (65.1%)	210 (43.3%)	<0.0001
Suspicious	756 (38.0%)	321 (30.7%)	160 (34.9%)	275 (56.7%)	
**Prostate volume**	52 (38, 70)	60 (45, 80)	48 (35, 61)	41 (32, 57)	<0.0001
**PSA density**	0.12 (0.08, 0.18)	0.10 (0.07, 0.14)	0.13 (0.09, 0.18)	0.17 (0.11, 0.26)	<0.0001
**Qmax, ml/s**	14 (10, 20)	13 (10, 19)	14 (10, 20)	15 (10, 23)	0.001
**PVR, ml**	30 (1, 50)	30 (1, 50)	20 (1, 40)	20 (1, 40)	<0.0001
**IPSS**	9 (5, 16)	10 (6, 17)	8 (4, 13)	8 (4, 15)	<0.0001
**Alpha blocker, n (%)**					
No	1288 (64.8%)	636 (60.9%)	306 (66.8%)	346 (71.3%)	0.0002
Yes	700 (35.2%)	409 (39.1%)	152 (33.2%)	139 (28.7%)	
**Irani G, n (%)**					
0-1	1349 (67.9%)	644 (61.6%)	350 (76.4%)	355 (73.2%)	<0.0001
2-3	639 (32.1%)	401 (38.4%)	108 (23.6%)	130 (26.8%)	
**Irani A, n (%)**					
0	1004 (50.5%)	449 (43.0%)	277 (60.5%)	278 (57.3%)	<0.0001
1-2-3	984 (49.5%)	596 (57.0%)	181 (39.5%)	207 (42.7%)	
**Irani Sum**					
0-1	951 (47.8%)	421 (40.3%)	268 (58.5%)	262 (54.0%)	<0.0001
2-3	797 (40.1%)	465 (44.5%)	139 (30.3%)	193 (39.8%)	
>3	240 (12.1%)	159 (15.2%)	51 (11.1%)	30 (6.2%)	

**Figure 1:**
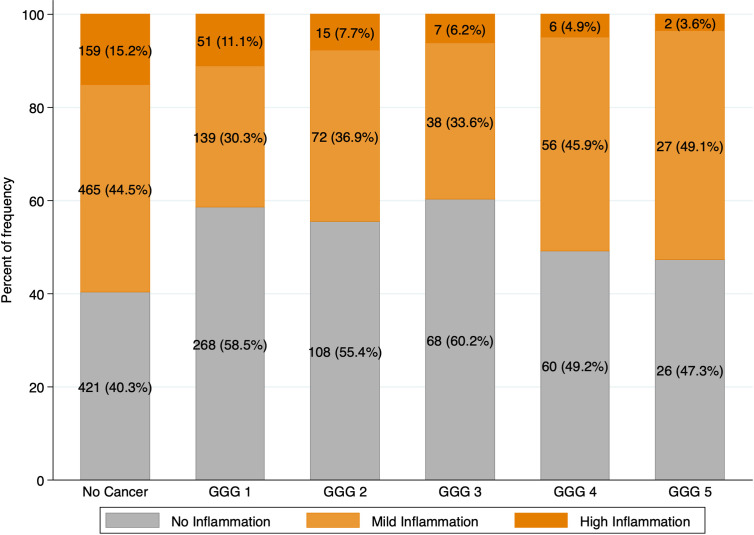
Intraprostatic inflammation according to Prostate Cancer Gleason Grade Groups. Intraprostatic inflammation was graded using Irani total score and categorized in three groups: no inflammation (Irani Sum 0-1); mild inflammation (Irani Sum 2-3); high inflammation (Irani Sum >3).

The distribution of mild (Irani total score 2-3) and high (Irani total score >3) prostatic inflammation according to GGG is graphed in **Figure 1** showing that these two conditions often coexist.

### Predictors of Prostatic Inflammation and csPCa

To evaluate specific predictors of prostatic inflammation (Irani score>1) we first divided the population in two groups based on the presence or absence of csPCa (**Table 2**). In the group without csPCa, patients with prostatic inflammation had a higher PSA (6.0 vs 5.0 ng/ml; p=0.0003), higher prostate volume (58 vs 52 cc; p<0.0001), were more likely to have a previous negative biopsy (29% vs 21%; p=0.0005) and a negative DRE (70% vs 65%; p=0.023) but no difference in PSA density (0.1 vs 0.11; p=0.2). Qmax, PVR and IPSS were slightly worse in patients with prostatic inflammation. Conversely in the group with csPCa, patients with prostatic inflammation had a higher prostate volume (43 vs 40 cc; p=0.007) but no difference in the other clinical parameters. At multivariable analysis adjusting for age, biopsy history and DRE, PSA density emerged as a strong predictor of csPCa (OR per 0.1 increase: 2.09; CI: 1.85, 2.35; p<0.001) but was not associated with prostatic inflammation (OR per 0.1 increase: 0.92; CI: 0.84, 1.01; p=0.073) (**Table 3**).

**Table 2:** Predictors of prostatic inflammation (Irani Score>1) in patients with and without csPCa.

	Negative Biopsy + GGG 1 PCa		P Value	csPCa (GGG≥2)		P Value
	IRANI Score 0-1	IRANI Score >1		IRANI Score 0-1	IRANI Score >1	
	689 (34.7%)	814 (40.9%)		262 (13.2%)	223 (11.2%)	
**Age**	66 (60, 70)	66 (60, 70)	0.5	70 (64, 75)	70 (65, 75)	0.3
**PSA**	5.5 (4.4, 7.8)	6.0 (4.6, 8.6)	**0.0003**	6.6 (4.7, 10.4)	7.0 (5.1, 10.8)	0.3
**Biopsy History, n (%)**						
Biopsy Naive	541 (78.5%)	575 (70.6%)	**0.0005**	237 (90.5%)	194 (87.0%)	0.2
Previous Neg.	148 (21.5%)	239 (29.4%)		25 (9.5%)	29 (13.0%)	
**DRE, n (%)**						
Negative	448 (65.0%)	574 (70.5%)	**0.023**	120 (45.8%)	90 (40.4%)	0.2
Suspicious	241 (35.0%)	240 (29.5%)		142 (54.2%)	133 (59.6%)	
**Volume, cc**	52 (40, 69)	58 (43, 80)	**<0.0001**	40 (30, 55)	43 (34, 60)	**0.007**
**PSA density**	0.11 (0.08, 0.16)	0.10 (0.07, 0.16)	0.2	0.18 (0.11, 0.26)	0.16 (0.11, 0.26)	0.3
**Qmax, ml/s**	14 (11, 21)	13 (10, 19)	**0.004**	16 (10, 25)	15 (10, 22)	0.3
**PVR, ml**	22 (1, 50)	30 (1, 50)	**0.005**	20 (1, 40)	20 (1, 40)	0.5
**IPSS**	9 (5, 16)	10 (5, 17)	**0.029**	8 (4, 15)	9 (5, 15)	0.2
**α blocker, n (%)**	242 (35.1%)	319 (39.2%)	0.10	70 (26.7%)	69 (30.9%)	0.3
**Bx GGG, n (%)**						
Negative	421 (61.1%)	624 (76.7%)	**<0.0001**	N/A	N/A	
GGG 1	268 (38.9%)	190 (23.3%)		N/A	N/A	

Bold means statistically significant.

N/A means not applicable.

**Table 3:** Univariable and Multivariable analysis to evaluate predictors of intraprostatic inflammation and clinically significant prostate cancer in the overall population (N=1988).

Covariate	Multivariable analysis predicting Intraprostatic inflammation			Multivariable analysis predicting csPCa		
	OR	95% CI	P>|z|	OR	95% CI	P>|z|
**Age, per y**	1.01	0.99,1.02	0.328	1.08	1.06,1.10	**<0.001**
**Biopsy History**						
Biopsy Naive	Ref.			Ref.		
Previous Neg.	1.55	1.25,1.92	**<0.001**	0.32	0.23,0.44	**<0.001**
**DRE**						
Negative	Ref.			Ref.		
Suspicious	0.85	0.71,1.03	0.097	2.21	1.76,2.78	**<0.001**
**PSA density, per 0.1**	0.92	0.84,1.01	0.073	2.09	1.85,2.35	**<0.001**

Intraprostatic inflammation was defined as Irani total score >1.

Bold means statistically significant.

### Histological Findings According to PSA Density

**Figure 2:**
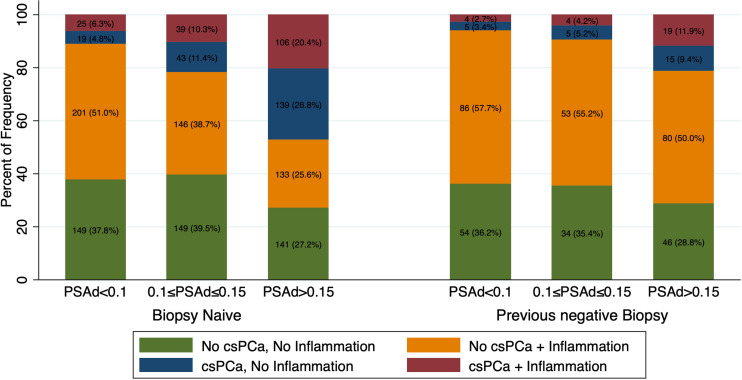
Bar graph showing frequency and rates of csPCa and Inflammation according to PSA density (PSAd) and biopsy history in patients with a PSA>4ng/ml (n=1694). Patients were stratified in four groups according to presence or absence of inflammation and csPCa: both present, both absent, intraprostatic inflammation without csPCa and csPCa without inflammation.

**Figure 2** graphically present histological findings of man who underwent prostate biopsy for a PSA >4 ng/ml (n=1694) according to biopsy history. Biopsy naïve patients with a PSA density below 0.1, were more likely to be diagnosed with prostatic inflammation (Irani total score >1) rather than csPCa (51% vs 11%, p <0.001). Conversely the rate of patients with csPCa was much higher with a PSA density between 0.10 and 0.15 (22%) and above 0.15 (47%). Similar results were found in patients with a previous negative biopsy, however rates of patients with csPCa were lower at each PSA density cut-off and resulted 6%, 9% and 21% in patients with a PSA density below 0.10, between 0.10 and 0.15 and above 0.15 respectively (all p <0.01). According to these findings, the optimal cutoffs of PSA density to diagnose csPCa and rule out the presence of prostatic inflammation in patients with an elevated PSA (>4 ng/ml) were 0.10 ng/ml^2^ in biopsy naïve patients and 0.15 ng/ml^2^ in patients with a previous negative biopsy.

**Figure 3:**
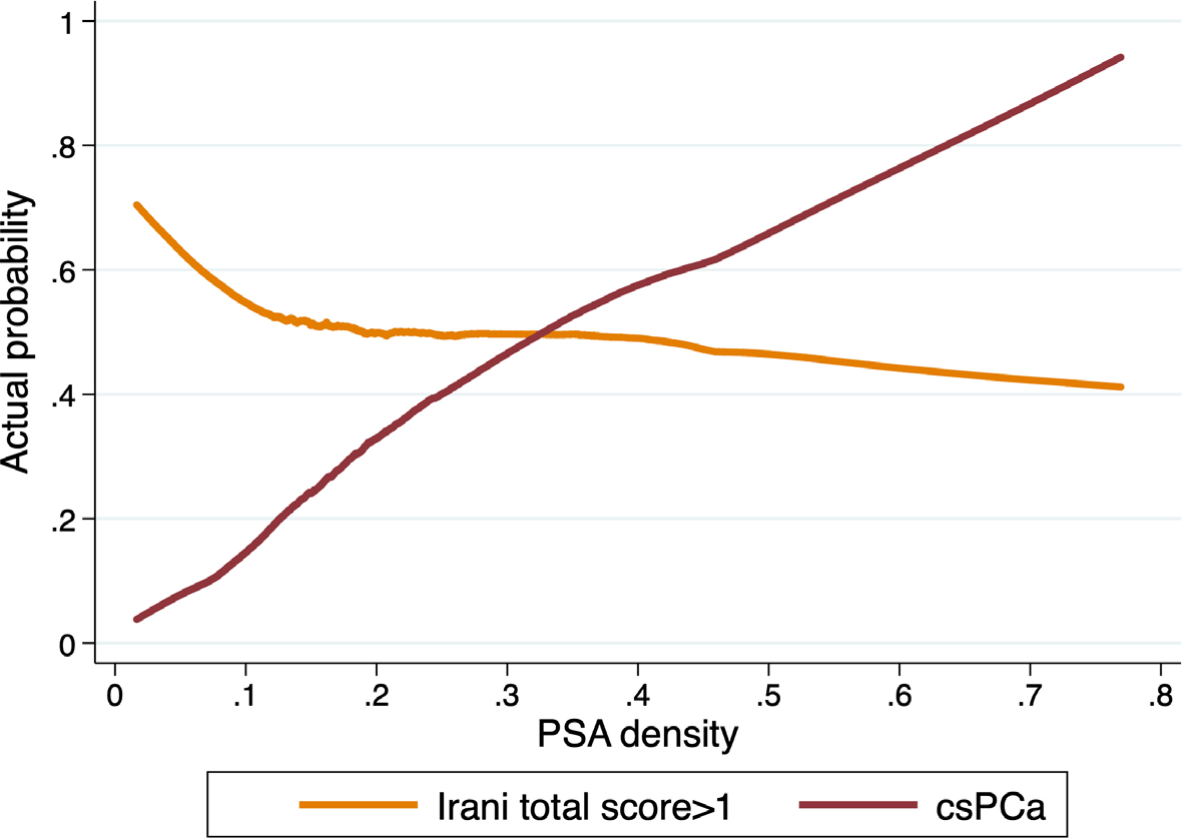
Actual probability of csPCa and prostatic inflammation (Irani score>1) in prostate biopsy samples according to PSA density in patients with PSA>4ng/ml (n=1694).

Using the locally weighted scatterplot smoothing method we further evaluated the association between PSA density, csPCa and prostatic inflammation. With increasing PSA density, the actual probability of csPCa increases while the likelihood of prostatic inflammation decreases (**Figure 3**).

## Discussion

A close correlation has been shown between prostate inflammation, BPH and csPCa.

The inflammatory process of the prostate through the release of cytokines and growth factors, promotes tissue injury, chronic immune response, and abnormal remodeling processes which can result in prostate enlargement and BPH as well as in malignant transformation of high proliferative cells (12).

In this scenario, several interesting findings emerged from our study. First of all, we found that prostatic inflammation and PCa are two conditions that often coexist. Although prostate tissue has been described in the past as an immunological desert, we found that patients with csPCa have moderate and severe inflammation in 30-50% and 5-10%, respectively.

The inflammatory process of the prostate through the release of cytokines and growth factors, promotes tissue injury, chronic immune response, and abnormal remodeling processes (12).

Preclinical studies provide a biological rationale for the association between inflammation and the risk of PCa, however clinical investigations report conflicting results. A recent meta-analysis of 25 studies involving a total of 20585 patients of whom 6641 with PCa demonstrated an inverse relationship between prostate inflammation on biopsy needle and malignant disease (6).

Similarly, in our previous publications we demonstrated that prostatic inflammation is a common finding in prostate biopsy samples, it is associated with benign prostatic obstruction rather than PCa (13) and can be used as a risk stratification tool in patients with a diagnosis of low to intermediate risk of PCa. Indeed, high grade inflammation was associated with a lower risk of upgrading and upstaging in patients undergoing radical prostatectomy (14). Since high grade prostatic inflammation is also associated with higher PSA levels and higher prostate volume, one of the possible explanations to these findings might be the role of prostatic inflammation as a confounding factor in the diagnosis of PCa. On the other side, prostatic inflammation may result in worse LUTS due to prostate enlargement and bladder outlet obstruction resulting in patient’s referral for urological evaluation. What we face here is the question of which comes first, the chicken or the egg. Either way prostatic inflammation and BHP parameters demonstrated an inverse correlation with PCa diagnosis (15–17) and with the present study we sought to determine the potential role of PSA density to rule out the presence of PI and benign disease in patients at risk of PCa. We found that PSA density is not affected by the presence of prostatic inflammation while, the actual probability of csPCa increases with increasing PSA density. Although this is, to the best of our knowledge, the first study focusing on PSA density and histologically confirmed prostatic inflammation, several studies corroborate our findings pointing out that PSA density outperform PSA alone in the prediction of csPCa. In a study including 1290 patients, Jue et al. showed that PSA density outperformed total PSA in the diagnosis of csPCa both in patients with a PSA in the “gray zone” (between 4 and 10 ng/ml) and in patients with PSA > 10 mg/ml. The difference in the predictive accuracy of PSA and PSA density was even higher in patients with a previous negative PBx (18).

What is the optimal cut-off of PSA density to suggest a prostate biopsy is still unclear. A PSA density cut-off of 0.15 ng/ml^2^ was suggested in previous studies (3). However, Nordström et al. showed that a PSA density cutoff of 0.10 and 0.15 ng/ml^2^ resulted in detection of only 77% and 49% of csPCa. Conversely, omitting prostate biopsy for men with PSA density ≤0.07 ng/ml^2^ would save 19.7% of biopsy procedures, while missing 6.9% of csPCa (19). In the present study, stratifying the population according to biopsy history, we showed that the optimal cutoffs of PSA density to rule out the presence of prostatic inflammation in patients with an elevated PSA (>4 ng/ml) were 0.10 ng/ml^2^ in biopsy naïve patients and 0.15 ng/ml^2^ in patients with a previous negative biopsy.

Still, PSA density it has not been incorporated into the early detection guidelines as a baseline measure because of the lack of precision of both PSA and prostate volume measurements using transrectal ultrasound.

MRI helped to overcome this limitation and recent studies pointed out that the combination of MRI parameters and PSA density could help to predict not only prostate biopsy results (20, 21), but also active surveillance outcomes (22), adverse pathologic features at RP (23) and biochemical recurrence after surgical treatment (24).

While several blood and urine biomarkers and imaging techniques have been developed to predict PCa (25, 26), as far as we know no biomarker is available for the diagnosis of prostate inflammation. At a time when immunotherapy is taking hold, the identification of cases with prostatic inflammation is of considerable interest for targeted immunological therapies (27).

The present study has few limitations. First, this is a single center study and histological evaluation was carried out by a single dedicated genitourinary pathologist. Even if the IRANI score is a validated score, a certain degree of interobserver variability may exist and limit the generalizability of our findings. Additionally, most patients underwent prostate biopsy without a prebiopsy MRI. The potential utility of MRI to rule out the presence of prostatic inflammation, as well as MRI diagnostic accuracy in patients with and without prostate inflammation should be further evaluated. Finally, we enrolled in the present study only patients in whom the clinical suspicion of PCa was deemed enough to perform PBx. While this may represent a potential source of inclusion bias, performing PBx in patients with low risk of PCa would be unethical.

Prostatic inflammation is a common cause of increased PSA. PSA density rather than PSA, should be used to evaluate patients at risk of prostate cancer who may need additional testing or prostate biopsy. This readily available parameter can potentially identify men who do not have PCa but have an elevated PSA secondary to benign conditions.

## Data Availability Statement

The raw data supporting the conclusions of this article will be made available by the authors, without undue reservation.

## Ethics Statement

The study was approved by the local Ethical Committee (Ethical Committee at the University Hospital “Ospedali Riuniti”, Foggia, Italy) and was carried out in agreement with the provisions of the Helsinki Declaration held in 1995. The patients/participants provided their written informed consent to participate in this study.

## Author Contributions

UF, SB, GB: study concept and design. Nd’A, FS, OS, VM: acquisition of data. VM, OS, UF, FG, MM: analysis and interpretation of data. UF: statistical analysis. SB, MR, GB: drafting and reviewing the paper. LC, GC, AS, EB, AP, MF, CB: supervision. All authors contributed to the article and approved the submitted version.

## Funding

This paper has been published with the financial support of the Dept. of Medical and Surgical Sciences of the University of Foggia.

## Conflict of Interest

The authors declare that the research was conducted in the absence of any commercial or financial relationships that could be construed as a potential conflict of interest.
